# Long-Term Consumption of Oats in Adult Celiac Disease Patients

**DOI:** 10.3390/nu5114380

**Published:** 2013-11-06

**Authors:** Katri Kaukinen, Pekka Collin, Heini Huhtala, Markku Mäki

**Affiliations:** 1 Department of Gastroenterology and Alimentary Tract Surgery, Tampere University Hospital, Tampere FIN-33521, Finland; E-Mail: pekka.collin@uta.fi; 2 School of Medicine, University of Tampere, Tampere FIN-33014, Finland; 3 Department of Medicine, Seinäjoki Central Hospital, Seinäjoki FIN-60220, Finland; 4 School of Health Sciences, University of Tampere, Tampere FIN-33014, Finland; E-Mail: heini.huhtala@uta.fi; 5 Center for Child Health Research, University of Tampere and Tampere University Hospital, Tampere FIN-33014, Finland; E-Mail: markku.maki@uta.fi

**Keywords:** celiac disease, gluten-free diet, morphology, oats, questionnaire, small-bowel

## Abstract

Many celiac disease patients tolerate oats, but limited data are available on its long-term consumption. This was evaluated in the present study, focusing on small-bowel mucosal histology and gastrointestinal symptoms in celiac adults maintaining a strict gluten-free diet with or without oats. Altogether 106 long-term treated celiac adults were enrolled for this cross-sectional follow-up study. Daily consumption of oats and fiber was assessed, and small-bowel mucosal morphology and densities of CD3+, αβ+ and γσ+ intraepithelial lymphocytes determined. Gastrointestinal symptoms were assessed by a validated Gastrointestinal Symptom Rating Scale questionnaire. Seventy (66%) out of the 106 treated celiac disease patients had consumed a median of 20 g of oats (range 1–100 g) per day for up to eight years; all consumed oat products bought from general stores. Daily intake and long-term consumption of oats did not result in small-bowel mucosal villous damage, inflammation, or gastrointestinal symptoms. Oat-consumers had a significantly higher daily intake of fiber than those who did not use oats. Two thirds of celiac disease patients preferred to use oats in their daily diet. Even long-term ingestion of oats had no harmful effects.

## 1. Introduction

Currently the only treatment for celiac disease is lifelong adherence to a strict gluten-free diet avoiding wheat-, rye-, and barley-derived prolamins. There is a large body of evidence to support the nutritional value [1,2,3] and safety of consumption of oats in the vast majority of both children and adults suffering from celiac disease and dermatitis herpetiformis [4,5,6,7]. There is, nevertheless, no consensus among scientists and nutrition experts as to whether oats can be unequivocally recommended for celiac patients [8,9,10]. One obvious concern is the safety of oats in long-term use. Even if products containing pure oats are nowadays on the market, many commercially available oat products are contaminated with wheat and barley during harvesting and milling processes [11,12]. Furthermore, some individuals may be intolerant even to pure oats, and case reports have shown that oat-intolerant celiac patients may have avenin-reactive T-cells in the small-bowel mucosa [13,14]. Oats has some wheat-like sequences in its protein structure, but as these are less frequent in oats than in other prolamins, it might take a considerably longer time to trigger a disease relapse [15]. Furthermore, limited data on the reasons for withdrawals among oat consumers in randomized trials implies some uncertainty [5,16,17]. Long-term follow-up studies on oats in celiac disease have been lacking, as the longest follow-up including small-bowel biopsies has been only five years [5].

Based on a statement from the scientific advisory board of the national Celiac Disease Society, consumption of oats has been allowed for celiac adults in Finland since 1997. The statement was extended in 1998 to apply to patients with dermatitis herpetiformis, and in 2000, also children. In consequence, about 70% of all celiac disease and dermatitis herpetifomis patients in Finland currently consume an oat-containing gluten-free diet [18]. In this cross-sectional study the aim was to assess the effects of long-term oat consumption on small-bowel mucosal villous morphology and inflammation and gastrointestinal symptoms in a series of celiac adults maintaining a strict gluten-free diet with or without oats.

## 2. Methods

### 2.1. Subjects

Altogether, 110 long-term treated celiac disease adults were invited to participate in a health survey comprising a follow-up small-bowel biopsy and clinical and dietary evaluation at the Department of Gastroenterology and Alimentary Tract Surgery, Tampere University Hospital, Finland. Patients found to be adhering to a strict gluten-free diet were eligible. Oat consumption was not an inclusion criterion. At diagnosis all patients had had biopsy-proven celiac disease, and after one year on a gluten-free diet clinical, serological or histological recovery was evident in all. The patients had been followed up in primary health care and no further routine small-bowel biopsies had been taken during the usual long-term surveillance. The study protocol was approved by the Ethical Committee of Tampere University Hospital. All subjects gave written informed consent.

### 2.2. Dietary Assessment

A detailed dietary analysis and a history of occasional or regular consumption of gluten-containing products, oats, and fiber were assessed by means of an interview by a trained dietician and by a four-day record of food intake [4]. The duration of oat intake was also recorded.

### 2.3. Small-Bowel Mucosal Morphology and Inflammation

Altogether, six small-bowel biopsy specimens were taken from the distal part of the duodenum upon esophago-gastroduodenoscopy; the specimens were evaluated by the same investigator without prior knowledge of history or findings. Three biopsies were formalin-fixed and embedded in paraffin; 5-μm-thick biopsy sections were stained with hematoxylin-eosin and studied under light microscopy. Morphometric analysis by measuring villous height and crypt depth ratio (Vh/CrD) was made in well-oriented biopsy samples as previously described [19], and Vh/CrD > 2 was considered normal. For immunohistochemical stainings, three biopsies were freshly embedded in optimal temperature compound (OCT, Tissue-Tec, Miles Inc., Elkhart, IN, USA), snap-frozen in liquid nitrogen, and stored at −70 °C. In 5-μm-thick frozen sections, CD3+ intraepithelial lymphocytes (IELs) were stained with monoclonal antibody Leu-4 (anti Leu-4 also known as anti CD3, Becton Dickinson, San Jose, CA, USA), αβ+ IELs with monoclonal βF1 antibody (Endogen, Woburn, MA, USA) and γδ+ IELs with TCRγ antibody (Endogen). IELs were counted with a 100× flat-field light microscope objective in randomly selected areas of surface epithelium and the density of IELs expressed as cells/millimeter of epithelium as previously described [19]. The reference values were set at 37 cells/mm for CD3+, at 25 cells/mm for αβ+, and at 4.3 for γδ+ IELs [19].

### 2.4. Gastrointestinal Symptoms and Clinical Evaluation

Gastrointestinal symptoms were evaluated by the Gastrointestinal Symptom Rating Scale (GSRS) questionnaire, which is also well-validated in celiac disease [17,20,21,22]. The questionnaire comprises, altogether, 15 items in five subdimensions describing: diarrhea (increased passage of stools, loose stools, urgent need for defecation), indigestion syndrome (borborygmus, abdominal distension, eructation, increased flatus), constipation (decreased passage of stools, hard stools, feeling of incomplete evacuation), abdominal pain (abdominal pain, nausea, and vomiting), and gastro-esophageal reflux (heart burn, acid regurgitation). Each item was graded from one to seven, a higher score indicating more gastrointestinal symptoms. In earlier studies, 95% confidence intervals of GSRS total scores have been 1.8–2.2 in non-celiac controls [22]. The body mass index (BMI) was calculated as weight in kilograms divided by the square of height in meters (normal range: 18.0–25.0 kg/m^2^).

### 2.5. Serology and Chemical Analysis

Serum IgA class endomysial antibodies (EmA) were determined using an indirect immunofluorescence method with human umbilical cord as substrate, and a dilution 1:≥5 was considered positive [4]. Serum IgA-class tissue transglutaminase antibodies (tTG-ab) were investigated by enzyme-linked immunosorbent assay (ELISA) (Celikey^®^, Phadia, GmbH, Freiburg, Germany); the result was classified as positive when ≥5.0 U/L. Blood hemoglobin level was measured using routine laboratory methods.

### 2.6. Statistics

Differences between patients consuming and avoiding oats were compared by Mann-Whitney U test or *t*-test, when appropriate. Data were given mainly as medians and range. Spearman’s coefficient was used for correlation studies. A *p*-value of <0.05 was considered statistically significant. All statistical testing was performed using SPSS version 16.0 (SPSS Inc., Chicago, IL, USA).

## 3. Results

At the beginning of the study, the dietician observed that four celiac disease patients had committed minor dietary transgressions less than once a month, and they were excluded from further studies. The remaining 106 treated celiac disease patients, adhering to a strict gluten-free diet, were deemed eligible. As shown in Table 1, altogether 70 (66%) out of the 106 treated celiac disease patients had preferred to consume oats in their otherwise strict gluten-free diet; 40 of them had taken oats for five years or more (up to eight years). All 70 patients used oat products purchased in local markets. The median daily intake of oats was 20 g, but 10 patients consumed 50–100 g per day. The clinical picture of celiac disease at diagnosis, age at study onset and the duration of the gluten-free diet were no different in patients who decided not to take oats from those favoring an oat-containing gluten-free diet, but oat consumers were more likely to have a family history of celiac disease (Table 1).

**Table 1:** Demographic data and dietary history in 106 treated celiac disease (CD) patients.

Characteristics		No Oats *n* = 36	Oats *n* = 70
Female (%)		25 (69%)	46 (66%)
Median age at time of study (range), years		54 (36–73)	59 (24–81)
Symptoms and signs leading to the diagnosis of CD, *n* (%)			
	Abdominal symptoms	29 (81%)	61 (87%)
	Malabsorption, anemia, loss of weight	17 (47%)	47 (67%)
	Dermatitis herpetiformis	4 (11%)	9 (13%)
	Extraintestinal symptoms ^a^	5 (14%)	11 (16%)
	Screening of risk groups ^b^	2 (6%)	4 (6%)
Family history of CD, *n* (%)		10 (28%)	42 (60%) ^c^
Median duration of gluten-free diet (range), years		10 (1–28)	8 (1–41)
Median duration of oat consumption after the diagnosis of CD (range), years		0	5 (0.5–8)
Median (range) daily intake of oats, g		0	20 (1–100)

^a^ Osteoporosis, arthritis, polyneuropathy, ataxia, mild memory disturbances, depression, anxiety, fatigue, fibromyalgia, enamel defects in permanent teeth, elevated liver enzymes; ^b^ Family history of CD, population screening; ^c^ *p* = 0.002 when compared to patients taking no oats; differences in sex, age, the difference between symptoms and duration of gluten-free diet was not statistically significant.

Altogether, 30 out of 36 subjects avoiding oats were diagnosed to have celiac disease before oat-containing gluten-free-products were permissible for adults with celiac disease; thus, after the diagnosis they all had been on a standard gluten-free diet without oats. Afterwards, one patients tried oats but he stopped using oats due to abdominal symptoms; the rest 29 did not want to start to consume oats again (partly do the fear of adverse effects). From the remaining six patients there were no data why they avoided oats.

**Figure 1:**
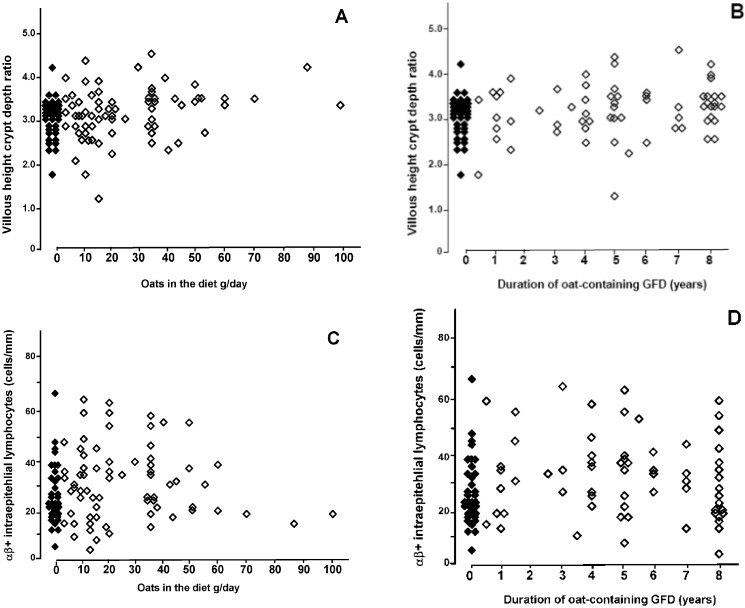
Small-bowel mucosal villous height crypt depth ratios in 106 treated celiac disease patients correlated with the daily oat intake (**A**) *r* = 0.251, *p* = 0.009 and the duration of oat consumption (**B**) *r* = 0.252, *p* = 0.012. Correlations between densities of αβ+ intraepithelial lymphocytes and daily oat intake (**C**), and between the cells and the duration of oat consumption (**D**) were not statistically significant (*r* = 0.152, *p* = 0.119 and *r* = 0.132, *p* = 0.190, respectively). GFD = gluten-free diet. Black diamond = no oats. Open diamond = oat user.

Small-bowel mucosal villous morphology was normal in 103 (97%) out of the 106 long-term treated celiac disease patients; two patients using, and one not using oats had abnormal villous structure. A high daily oat intake and a long duration of oat intake correlated with a better small-bowel mucosal Vh/CrD ratio (Figure 1A,B). The densities of mucosal αβ+ IELs were not different between patients who did or did not consume oats (Figure 1C,D). Similarly, small-bowel mucosal CD3+ and γσ+ IEL counts did not correlate with the daily oat intake (*r* = 0.134, *p* = 0.170 and *r* = 0.167, *p* = 0.088, respectively) or the duration of oat consumption (*r* = 0.029, *p* = 0.773 and *r* = 0.043, *p* = 0.771, respectively).

**Figure 2:**
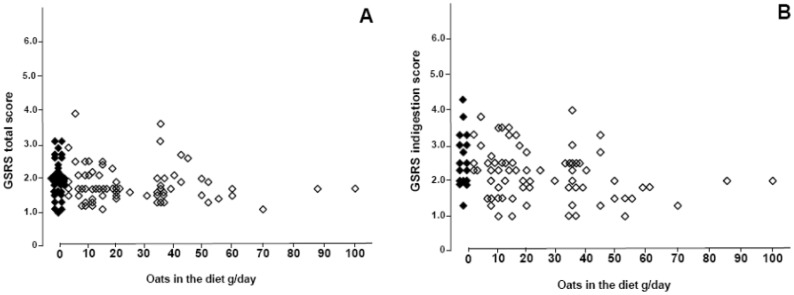
Gastrointestinal symptom rating scale (GSRS) total score correlated negatively with daily oat intake in 106 treated celiac disease patients (**A**) *r* = −0.220, *p* = 0.025, but not with the duration of oat consumption (*r* = −0.166, *p* = 0.101 [data not shown]). A high daily oat intake and a long duration of oat intake correlated with less indigestion (**B**) *r* = −0.313, *p* = 0.003 and *r* = −0.232, *p* = 0.037 [data not shown], respectively. Black diamond = no oats. Open diamond = oat user.

Based on GSRS total score, oat-consumers did not suffer more gastrointestinal symptoms than non-oat consumers (Figure 2A). In GSRS subdimensions, a higher oat intake and a long duration of oat intake correlated significantly with fewer complaints of indigestion (Figure 2B); there were no significant differences in other subdimensions (data not shown). Daily fiber intake was significantly higher in celiac disease patients who consumed oats than in those who did not (*p* = 0.013; median 18.1 g, range 7.6–50.9 g *vs.* median 15.8 g, range 6.0–23.3 g). None of the 13 patients with dermatitis herpetiformis suffered from cutaneous rash involvement; nine of the 13 were taking oats. BMI was no different between those treated celiac disease patients who did or did not consume oats (median 25.5 kg/m^2^, range 19.2–33.0 kg/m^2^ *vs.* 26.6 kg/m^2^, range 19.0–34.6 kg/m^2^ *p* = 0.671). All treated celiac patients were negative for serum EmA- and tTG-abs (median 0.5 U/L, range 0–2.9 U/L). Blood hemoglobin levels did not correlate with the consumption of oats (data not shown).

## 4. Discussion

In this large cross-sectional study we showed that in celiac disease long-term consumption of oats for up to eight years had no detrimental effect on symptoms, small-bowel mucosal villous morphology and inflammation, or on humoral response against tissue transgluatminase. In contrast, the mucosal morphology was even significantly better in subjects who had consumed oats in larger amounts or over a longer time-period than in those who did not take oats (Figure 1A,B). Our data confirm the results of an earlier five-year follow-up study in 23 celiac disease adults showing that the long-term ingestion of oats is safe [5].

Some studies have indicated that there is a subgroup of celiac patients who experience gastrointestinal symptoms more frequently on an oat-containing gluten-free diet than on a gluten-free diet without oats [4,14,16,17]. Flatulence and abdominal distension often occur soon after commencing an oat-containing diet, but in most cases the symptoms disappear gradually as consumption of oats continues [1]. This has been explained by an increased intake of fiber in oat products. Indeed, many non-celiac individuals develop similar symptoms when they suddenly start to consume oats [23]. In the current study celiac disease patients consuming oats, even up to 100 g per day, experienced no more gastrointestinal symptoms than those taking less or avoiding oats. Interestingly, patients who ingested high amounts of oats and for longer periods experienced less indigestion (Figure 2). In our series there were no oat-intolerant celiac disease patients who had previously consumed oats but discontinued due to symptoms. According to the literature, such patients may exist, but usually the occurrence of symptoms has not been associated with small-intestinal mucosal damage or inflammation [4,17]. Earlier, a Norwegian research group demonstrated that some oat-intolerant celiac disease patients have oat avenin-reactive T-cells in the small-intestinal mucosa [13,14]. Altogether, it would appear that even if most celiac patients tolerate oats, there might be some who have to avoid it in order to maintain remission. Interestingly, it has recently been shown that oat immunogenicity may vary between different oat cultivars [24]. In the current study it was impossible to trace which cultivars were used, as the patients were able to use a wide range of commercial oat products from the market. This notwithstanding, the patients remained in clinical and histological remission.

In the current study all celiac disease patients on an oat-containing gluten-free diet consumed ordinary oat products bought from general stores and meant for normal consumption. It has recently been shown that most commercially available oat products in both Europe and the United States are contaminated with wheat or barley, gluten levels ranging even from 200 to 8000 mg/kg (=ppm) when measured by the R5 antibody-based ELISA method [11,12]. The inevitable question is how to achieve a good clinical and histological response when consuming potentially gluten-contaminated oats. Firstly, in the long run, patients choose to consume relatively small doses of oats daily. If the product in question were contaminated even with 1000 ppm gluten, a daily consumption of 20 g oats would have resulted in no more than 20 mg gluten intake per day, an amount evidently tolerated by most celiac patients [25,26]. Secondly, according to earlier studies many oat products have been contaminated with barley [11]. There are only a few clinical studies on the toxicity of barley in celiac disease. Thirdly, the R5-ELISA method may overestimate barley contamination in oats, implying that in fact the oat products in question may have much lower contamination levels than reported [27]. It must also be considered that naturally gluten-free products, such as rice, corn, buckwheat, and soy may also be similarly contaminated with gluten [26,28]. Furthermore, there are studies showing that only 20%–73% of long-term treated patients adhering to a strict naturally gluten-free diet without oats evinced normal small-bowel mucosal villous architecture [29,30]. This implies that inadvertent gluten ingestion is not restricted to oats, but is also possible when consuming only products gluten-free by nature. Furthermore, it must be noted that a large number of long-term treated celiac disease patients had high numbers of αβ+ IELs, irrespective of oats ingestion. Persistent small-bowel mucosal intraepithelial lymphocytosis proved here again to be a common finding in well-treated celiac disease patients [29,31]. Interestingly, our recent study suggested that oats might contribute to the duodenal lymphocytosis [31]. Nevertheless, when in that study, a lower cut of value 25 IELs per 100 enterocytes was used for defining intraepithelial lymphocytosis, no association between oat consumption and lymphocytosis was found [31]. Altogether, compared to the findings in the earlier studies the patients in the present study had shown an excellent response to a strict gluten-free diet, even though they had consumed commercial oats for years. As pure oat products are nowadays available, consumption of ordinary, non-dedicated oat products (having potential risk of being contaminated by gluten) is discouraged, especially if a celiac disease patient decides to consume high amounts of oats daily.

## 5. Conclusions

To conclude, long-term consumption of oats proved to be safe for celiac disease patients. Oats diversifies a gluten-free diet, and enhances its nutritional quality by increasing the intake of dietary fiber. When allowed, most celiac disease patients in this country prefer to consume some oats. Pure oat products with strictly controlled production systems are nowadays available endorsing their use more widely. Long-term regular follow-up of celiac disease patients is recommended; those using oats may safely be followed up similarly to non-users.
